# Does the Location of Oral Lesions in Oral Lichen Planus Allow Predicting the Outcome of Its Histopathological Examination?

**DOI:** 10.3390/jcm14124076

**Published:** 2025-06-09

**Authors:** Katarzyna Osipowicz, Emilia Milczarek, Renata Gorska, Cezary Kowalewski, Janusz Pach, Piotr Regulski, Katarzyna Wozniak

**Affiliations:** 1 Department of Dermatology, Medical University of Warsaw, 02-008 Warsaw, Poland; 2 OT.CO Clinic, 00-716 Warsaw, Poland; 3 Department of Mucosal and Periodontal Diseases, University Stomatology Center of the Medical University of Warsaw, 02-097 Warsaw, Polandrenata.gorska@wum.edu.pl (R.G.); 4 Department of Dermatology, National Medical Institute of the Ministry of the Interior and Administration, 02-507 Warsaw, Poland; czarekkowalewski@gmail.com (C.K.);; 5 Laboratory of Digital Imaging and Virtual Reality, Department of Dental and Maxillofacial Radiology, 02-097 Warsaw, Polandpiotr.regulski@wum.edu.pl (P.R.)

**Keywords:** oral lichen planus, location, lesions, histopathology, diagnostic value, mouth ulcers

## Abstract

**Objective:** Numerous reports describe the typical localization of lesions in the oral cavity in various conditions, manifesting as oral lesions. We aimed to evaluate the correlation between these lesions and the results of histopathological examinations (HP). **Methods**: We examined 66 consecutive patients with oral lesions consistent with the clinical presentation of oral lichen planus (OLP). Standard HP evaluation was performed using light microscopy and, subsequently, the association between the precise location of the lesion and the histopathological diagnosis was assessed. **Results**: Erosions on the mandibular gingiva (*p* = 0.0086), white patches on the right buccal mucosa (*p* = 0.03197), white patches on the ventral surface of the tongue (*p* = 0.0397), white patches on the maxillary gingiva (*p* = 0.0228), white patches on the mandibular gingiva (*p* = 0.0062), white patches on the upper lip (*p* = 0.0226), and bilateral white patches on the buccal mucosa (*p* = 0.0104) were significantly more prevalent in patients with positive histopathological findings. White patches on the buccal mucosa emerged as the strongest predictor of histopathological diagnosis of OLP, with a sensitivity of 91.3% and a negative predictive value of 85.7%. **Conclusions**: The location of oral lesions may indicate OLP with high sensitivity and a negative predictive value, which may facilitate further diagnostic management; however, it is not sufficient to establish a definitive diagnosis.

## 1. Introduction

Oral ulcerations are non-specific symptoms of oral lichen planus (OLP) since they appear in the course of many different diseases. The prevalence of oral ulcers varies across populations and is influenced by numerous factors, such as age, gender, geographic location, overall health, and lifestyle (including stress, tobacco use, and dietary supplement intake, including vitamins). Direct causes include adverse reactions to drugs, infections, inflammatory diseases, and oral cancer [1]. Diagnosing oral ulcers poses several challenges due to the nonspecific symptoms and underlying conditions, ranging from trivial to life-threatening. Proper history and examination usually direct to other specialists. Some of the highly alarming symptoms and signs include a history of bone marrow or organ transplant (may suggest oral manifestation of graft versus host disease or side effects of immunosuppressive therapies), skin lesions (which may suggest a plethora of dermatological diseases), enlarged lymph nodes (occurring in systemic diseases and malignancies), or a single, non-painful induration, suggesting cancer.

Although histopathological examination remains the primary test in the diagnostics of oral lesions, there is some correlation between their number, visual characteristics, location in the oral cavity, and a final diagnosis. Awareness of the relationship between the clinical picture and the histopathology result is particularly useful at the first visit, as it allows the clinician to interview the patient in an appropriate manner so as to neither cause unnecessary panic nor allow the patient to disregard further diagnostic recommendations [2].

Although Bacci et al. showed that dentists’ clinical diagnoses were erroneous (compared to HP) in 31.5% of cases [3], there are few reports on the correlation between the clinical picture and histopathological features in OLP. Several prior studies on agreement between clinical and pathologic diagnoses disclosed concordance rates ranging from 50% to over 80% [4,5,6,7,8]. In an Iranian retrospective study of 3001 patients, 2167 cases (72.2%) were consistent between clinical and histopathologic diagnoses [9]. Location and type of lesion (five groups based on the clinical manifestations) were indicated to have a significant effect on diagnosis. In regard to location, the highest concordance between clinical and histopathologic diagnoses was observed in mouth floor lesions (85.2%), buccal mucosa (83.3%), lip (82.7%), and labial mucosa (82.1%), whereas the lowest one was in gingival mucosa (66.1%). In terms of lesion category, the highest and the lowest concordance rates belonged to white and red lesions (86.1%) and pigmented lesions (47.1%), respectively [9]. A Spanish team retrospectively studied a group of 59 adult patients with confirmed clinical and histopathological diagnosis of OLP [10]. In this research, the buccal mucosa was the most frequent site for lesions, with 33.9% of cases exclusively affecting this area, but the tongue and gingiva were subsequent in prevalence. Despite that, location was not related to any clinical or histopathological features. A recent Japanese study on 132 patients with OLP showed the buccal mucosa as the most affected site (71.7%) and the reticular form was the most common clinical feature (n = 124). Biopsy was performed in 48 patients and the erosive form was the most common among the biopsied lesions (33%). In 77 (58.3%) patients, the lesions were located bilaterally [11]. Another study showed that the involvement of buccal mucosa (55.6%) followed by the tongue (7.8%) and labial mucosa (7.8%) were the most common locations [12]. Reticular OLP (53.3%) was the most common morphological type, followed by erosive OLP (23.3%). Clinicopathological correlation was achieved in 65.6% of the cases.

Several other studies showed that OLP typically has a bilateral distribution and most commonly appears on the buccal mucosa, tongue, and gingiva, followed by the labial mucosa and the lower lip [13,14,15,16]. Approximately 10% of OLP patients present with lesions confined to the gingiva, appearing as desquamative gingivitis [17]. The Koebner phenomenon, where the lesions develop at sites with mechanical trauma (e.g., friction from rough dental restorations/teeth or lip/cheek biting), may explain why the lesions appear more commonly on the buccal mucosa and tongue, which are more prone to trauma [18].

All of the authors cited above described their data using descriptive statistics. They did not calculate the basic parameters commonly used to assess the quality of a test. Therefore, the aim of our study was a quantitative evaluation of the correlation between the location of the lesion and the features of OLP assessed in histopathological examinations, determining the sensitivity, specificity, positive and negative predictive values, and overall diagnostic accuracy. We focused on the location of the lesions in view of the fact that our initial analysis showed a lack of predictive power for other features of such lesions [19].

## 2. Material and Methods

Our cohort study was of an observational nature. Patients participating in this study were referred by dentists and other dermatologists for the differentiation of mucosal lesions and, subsequently, diagnosed and treated as a part of routine medical care at the Department of Dermatology between 2019 and 2023. All patients provided informed consent for the performed medical procedures and, due to standard diagnostic procedures, approval from the ethics committee was not required. The protocol included a medical history, physical examination, and standard histopathology. Sections of oral mucosa lesions were obtained from patients using a 0.4 cm rotary barb from the edge of the oral lesion under local anesthesia with xylocaine. Subsequently, the sections were placed in formalin and transported to the laboratory. Histopathology was performed according to routine procedures—the examination was conducted by a qualified laboratory technician and approved by a laboratory diagnostician. Van der Waal’s criteria [20] were used for the diagnosis of OLP. The collected data were described using descriptive statistics (frequency and percentage) and a contingency table was created. The distribution was compared using the chi-square test (Statistica 13, Tibco, Tulsa, OK, USA) with the significance level set at 0.05. Significance levels between 0.05 and 0.1 were interpreted as trends toward dependence. Diagnostic test quality indicators were calculated using the Diagnostic Test Evaluation Calculator (MedCalc Software, online calculator, available from https://www.medcalc.org/calc/rate_ci.php accessed on 7 May 2025) and expressed as percentages with 95% confidence intervals.

## 3. Results

In this investigation, 66 participants took part, comprising 17 males (25.8%) and 49 females (74.2%), all of whom presented with oral manifestations indicative of OLP (Table 1). Among them, 51 (91.1%) displayed erosions and 51 (91.1%) exhibited ulcerations. Notably, 36 patients (62.0%) had bilateral erosions on the buccal mucosa, a characteristic feature of OLP, while 39 (73.4%) showed bilateral white patches on the buccal mucosa. In our analyzed cohort, only 33 patients (50.0%) received a histopathological confirmation of the diagnosis of OLP.

Erosions on the mandibular gingiva (*p* = 0.0086), white patches on the right buccal mucosa (*p* = 0.03197), white patches on the ventral surface of the tongue (*p* = 0.0397), white patches on the maxillary gingiva (*p* = 0.0228), white patches on the mandibular gingiva (*p* = 0.0062), white patches on the upper lip (*p* = 0.0226), and white patches bilaterally on the buccal mucosa (*p* = 0.0104) were significantly more frequent in patients with a positive histopathology. In addition, erosions on the ventral surface of the tongue (*p* = 0.0580), erosions on the upper lip (*p* = 0.0870), and white patches on the left buccal mucosa (*p* = 0.0590) showed a trend toward significance (Table 2). With the exception of white patches on the buccal mucosa, all other features showed a negative association with the histopathological diagnosis of OLP. As a result, white patches on the buccal mucosa were the relatively best predictor of histopathological diagnosis of OLP, with a sensitivity of 91.3% (95% confidence interval [CI] 71.96–98.93) and a negative predictive value of 85.7% (95% CI 59.79–96.03) (Table 3).

## 4. Discussion

Our analysis showed an association between the location of mucosal lesions and the results of the histopathologic examination. Among the analyzed locations of lesions, statistically significant or very closely significant associations with the histopathological diagnosis of OLP were found for erosions located on the ventral surface of the tongue, on the mandibular gingiva, the upper lip, white patches on the left buccal mucosa, white patches on the right buccal mucosa, white patches bilaterally on the buccal mucosa, white patches on the ventral surface of the tongue, white patches on the maxillary gingiva, white patches on the mandibular gingiva, and white patches on the upper lip. Among our examined patients, none with an erosion or white patch located on the ventral surface of the tongue or on the upper lip, or with white patches on the maxillary or mandibular gingiva, had a positive histopathological result for OLP. Such a location suggests a different nature of the lesion. This is a surprising discovery, as many review articles often indicate these locations of lesions as possible in this diagnosis [21,22,23]. The most likely explanation for the absence of patients in our sample diagnosed with OLP and lesions on the gums or on the ventral surface of the tongue is the size of our cohort. To verify this hypothesis, we calculated retrospectively the statistical power for the proportions in our sample, using a comparative group of 563 patients diagnosed with OLP from Croatia [24]. In this population, involvement of the floor of the mouth was recorded in 2.5%, translating to just under 15% power in our sample. This precludes drawing conclusions about a statistically significantly lower frequency of floor of the mouth involvement in our population compared to the approximately 5% reported in other studies from various countries [25,26,27,28,29]. The calculation of power for other locations compared to the population closest genetically to Poles was not possible due to the lack of distinction between maxillary and mandibular gums as well as the absence of differentiation between erosions and white patches.

Our results are similar to those published by Feldmeyer et al. in 2020, who also demonstrated a significant association between histopathological outcome and bilateral involvement of the buccal mucosa [30]. In their study, also monocentric and conducted in real-life settings, bilateral changes in the buccal mucosa were present in 87.2% of cases of OLP and 58.6% of cases of oral lichenoid lesions (OLL), while unilateral changes occurred in 8.1% of OLP cases and 24.1% of OLL cases. No such changes were observed in 4.7% of OLP cases and 17.2% of OLL cases. However, in this study as well, the sensitivity and specificity of such conclusions were not calculated. Moreover, our findings detail quantitatively previous literature reports indicating that the bilateral involvement of buccal mucosa is the most common. We found that the most significant diagnostic value was attributed to the presence of ulcers on the mucous membrane of the cheeks. In terms of negative predictive value (NPV), this symptom significantly surpasses even the presence of fibrinogen deposits detected by the direct immunofluorescence (DIF) method (88.89% vs. 57.78% and 56.25%) and, in terms of positive predictive value (PPV), it only slightly lags behind (50.0% vs. 66.67%), clearly distinguishing itself from other locations of ulcers and lesions that we described previously [31].

Our results indicate that a diagnosis cannot be established solely based on the lesion’s location (histopathology remains essential); however, this parameter can be used to quantitatively estimate the probability of the disease. This is particularly important in cases where a histopathological examination yields inconclusive results, when the patient refuses to consent to a biopsy, or when contraindications prevent the procedure. OLP remains a diagnostically complex disease, with diagnosis relying on multiple factors derived from patient history and laboratory tests. Lesion location serves as a supportive criterion, but previously proposed diagnostic criteria remain in place [20]. While our findings require further validation on larger cohorts, they provide a direction for assessing diagnostic uncertainty. The advantage of such quantitative tools is that they enable both physicians and patients to make more informed therapeutic decisions. Efforts to develop new diagnostic criteria are ongoing but have not led to a breakthrough. Recently, authors of a systematic review on OLP diagnostic methods proposed a new scoring system that incorporates lesion location—an innovation compared to previous systems [32]. However, they did not present quantitative data on the classification model’s reliability; hence, a reliable scoring model for this disease is still missing. Another study demonstrated that the diagnostic criteria proposed by the American Academy of Oral and Maxillofacial Pathology are not equivalent to WHO criteria [33], indicating that these criteria do not resolve the existing diagnostic challenges of OLP.

A limitation of our study is its sample size and recruitment from only one center. Therefore, the findings may not generalize to broader populations. The study was not designed to test hypotheses but rather to describe a sample collected in clinical practice. As a result, it did not have inclusion criteria, which may result in selection bias. The sample size was not estimated, and post hoc power analysis was not feasible due to the lack of a method for this type of endpoint. Due to the exploratory nature of the study, Bonferroni correction for multiple comparisons was not applied.

However, even such a study demonstrates that under the conditions of everyday clinical practice, with numerically limited populations available to a single doctor, one should exercise extreme caution when making inferences about the nature of a lesion based solely on its location.

## 5. Conclusions

The bilateral localization of white patches on the buccal mucosa is a factor that strongly supports the diagnosis of OLP. We believe that, in line with the guidelines [34], a clinical diagnosis must be supported by a histopathological examination—particularly due to its malignant potential, which, though low, cannot be ignored [35]—as well as by DIF analysis, despite the fact that this test may not always provide a definitive conclusion [36]. Further research on the etiology and pathogenesis of OLP is necessary, as well as the development of new diagnostic methods and multifactorial classification models to facilitate a differential diagnosis of this disease.

## Figures and Tables

**Table 1:** Baseline characteristic of the patients.

Feature	Value
Sex: male N (%)	17 (25.8)
Age at onset, median (interquartile range)	59.5 (40–64)
Months since onset, average (SD)	91.0 (97.2)
Smokers N (%)	3 (4.5)

**Table 2:** The number and percentage of patients with positive or negative results of histopathologic examination among those with lesions observed in specific locations. Concordance rate between location and histopathology is marked with *.

N, %	HP		*p*-Value
	Positive	Negative	
Erosions, any location Yes, n = 51 (91.1) No, n = 5 (8.9)	23 (45.1 *)	28 (54.9)	0.5237
	3 (60.0)	2 (40.0)	
Erosions on palate Yes, n = 13 (24.1) No, n = 41 (75.9)	6 (46.15 *)	7 (53.8)	0.9906
	19 (46.3)	22 (53.7)	
Erosions on left buccal mucosa Yes, n = 38 (67.9) No, n = 18 (32.1)	20 (52.63 *)	18 (47.4)	0.1763
	6 (33.3)	12 (66.7)	
Erosions on right buccal mucosa Yes, n = 41 (73.2) No, n = 15 (26.8)	19 (46.3 *)	22 (53.7)	0.9828
	7 (46.7)	8 (53.3)	
Erosions bilaterally on buccal mucosa Yes, n = 36 (62.0) No, n = 22 (38.0)	19 (52.8 *)	17 (47.2)	0.2240
	8 (36.4)	14 (63.6)	
Erosions on the tonque Yes, n = 8 (14.6) No, n = 47 (85.4)	5 (62.5 *)	3 (37.5)	0.3507
	21 (44.7)	26 (55.3)	
Erosions on ventral surface of the tonque Yes, n = 4 (7.3) No, n = 51 (92.7)	0 (0.0 *)	4 (100.0)	**0.0580**
	25 (49.0)	26 (51.0)	
Erosions on maxillary gingiva Yes, n = 20 (35.7) No, n = 36 (64.3)	8 (40.0 *)	12 (60.0)	0.4722
	18 (50.0)	18 (50.0)	
Erosions on mandibular gingiva Yes, n = 16 (28.6) No, n = 40 (71.4)	3 (18.7 *)	13 (81.3)	**0.0086**
	23 (57.5)	17 (42.5)	
Erosions on upper lip Yes, n = 3 (8.3) No, n = 33 (91.7)	0 (0.0 *)	3 (100.0)	**0.0870**
	17 (51.5)	16 (48.5)	
Erosions on lower lip Yes, n = 5 (11.1) No, n = 32 (88.9)	1 (25.0 *)	3 (75.0)	0.3450
	16 (50.0)	16 (50.0)	
White patches, any location Yes, n = 51 (96.2%) No, n = 2 (3.8%)	22 (43.1 *)	29 (56.9)	0.8477
	1 (50.0)	1 (50.0)	
White patches on palate Yes, n = 6 (11.3) No, n = 47 (88.7)	2 (33.3 *)	4 (66.7)	0.5974
	21 (44.7)	26 (55.3)	
White patches on left buccal mucosa Yes, n = 42 (79.2) No, n = 11 (20.8)	21 (50.0 *)	21 (50.0)	**0.0590**
	2 (18.2)	9 (81.8)	
White patches on right buccal mucosa Yes, n = 44 (83.0) No, n = 9 (17.0)	22 (50.0 *)	22 (50.0)	**0.03197**
	1 (11.1)	8 (88.9)	
White patches bilaterally on buccal mucosa Yes, n = 39 (73.4) No, n = 14 (26.4)	21 (53.8 *)	18 (46.2)	**0.0104**
	2 (14.3)	12 (85.7)	
White patches on the tongue Yes, n = 4 (7.5) No, n = 49 (92.5)	2 (50.0 *)	2 (50.0)	0.7817
	21 (42.9)	28 (57.1)	
White patches on ventral surface of the tongue Yes, n = 5 (9.4) No, n = 49 (90.6)	0 (0.0 *)	5 (100.0)	**0.0397**
	23 (47.9)	25 (52.1)	
White patches on maxillary gingiva Yes, n = 6 (11.3) No, n = 47 (88.7)	0 (0.0 *)	6 (100.0)	**0.0228**
	23 (48.9)	24 (51.1)	
White patches on mandibular gingiva Yes, n = 8 (15.4) No, n = 44 (84.6)	0 (0.0 *)	8 (100.0)	**0.0062**
	23 (52.3)	21 (47.7)	
White patches on upper lip Yes, n = 5 (13.9) No, n = 31 (86.1)	0 (0.0 *)	5 (100.0)	**0.0226**
	17 (54.8)	14 (45.1)	
White patches on lower lip Yes, n = 6 (17.7) No, n = 28 (82.3)	1 (16.7 *)	5 (83.3)	0.1356
	14 (50.0)	14 (50.0)	

Text in bold indicates statistical significance. The *p*-value was obtained from the chi-square test.

**Table 3:** The reliability of parameters of lesion location in establishing the diagnosis of oral lichen planus in relation to the histopathologic examination results as the reference test.

	Erosions on Ventral Surface of the Tongue		Erosions on Mandibular Gingiva		Erosions on Upper Lip		White Patches of Left Buccal Mucosa		White Patches on Right Buccal Mucosa		White Patches Bilaterally on Buccal Mucosa		White Patches on Ventral Surface of the Tongue		White Patches on Maxillary Gingiva		White Patches on Mandibular Gingiva		White Patches on Upper Lip	
Statistic	Value	95% CI	Value	95% CI	Value	95% CI	Value	95% CI	Value	95% CI	Value	95% CI	Value	95% CI	Value	95% CI	Value	95% CI	Value	95% CI
Sensitivity	0.00	0.00–13.72	11.54	2.45–30.15	0.00	0.00–19.51	48.84	33.31–64.54	95.65	78.05–99.89	91.30	71.96–98.93	0.00	0.00–14.82	0.00	0.00–14.82	0.00	0.00–14.82	0.00	0.00–19.51
Specificity	86.67	69.28–96.24	56.67	37.43–74.54	84.21	60.42–96.62	51.16	35.46–66.69	26.67	12.28–45.89	40.00	22.66–59.40	83.33	65.28–94.36	80.00	61.43–92.29	70.00	50.60–85.27	73.68	48.80–90.85
Positive Predictive Value	0	0	18.75	6.87–41.92	0	0	50.00	39.35–60.65	50.00	44.21–55.79	53.85	45.91–61.59	0	0	0	0	0	0	0	0
Negative Predictive Value	50.98	47.47–54.48	42.50	34.42–51.00	48.48	43.65–53.35	50.00	39.82–60.18	88.89	51.82–98.35	85.71	59.79–96.03	52.08	48.08–56.06	51.06	46.60–55.51	47.73	41.94–53.58	45.16	38.63–51.86
Accuracy	47.27	33.65–61.20	35.71	23.36–49.64	44.44	27.94–61.90	50.00	39.02–60.98	56.60	42.28–70.16	62.26	47.89–75.21	47.17	33.30–61.36	45.28	31.56–59.55	39.62	26.45–54.00	38.89	23.14–56.54

Sensitivity: Probability that a test result will be positive when the disease is present (true positive rate). Specificity: Probability that a test result will be negative when the disease is not present (true negative rate). Positive predictive value: Probability that the disease is present when the test is positive. Negative predictive value: Probability that the disease is not present when the test is negative. Accuracy: Overall probability that a patient is correctly classified.

## Data Availability

Additional data are available upon reasonable request, after anonymization, due to privacy restrictions.
